# RNase alleviates neurological dysfunction in mice undergoing cardiac arrest and cardiopulmonary resuscitation

**DOI:** 10.18632/oncotarget.18088

**Published:** 2017-05-23

**Authors:** Ye Ma, Chan Chen, Shu Zhang, Qiao Wang, Hai Chen, Yuanlin Dong, Zheng Zhang, Yan Li, Zhendong Niu, Tao Zhu, Hai Yu, Bin Liu

**Affiliations:** ^1^ Department of Anesthesiology and Translational Neuroscience Center, West China Hospital, Sichuan University, Chengdu, Sichuan, China; ^2^ Department of Emergency Medicine, West China Hospital, Sichuan University, Chengdu, Sichuan, China; ^3^ Geriatric Anesthesia Research Unit, Department of Anesthesia, Critical Care and Pain Medicine, Massachusetts General Hospital and Harvard Medical School, Boston, MA, USA

**Keywords:** RNase, cardiopulmonary resuscitation, neurological outcome, inflammation, autophagy

## Abstract

Cardiac arrest (CA) is one of the leading lethal factors. Despite cardiopulmonary resuscitation (CPR) procedure has been consecutively improved and lots of new strategies have been developed, neurological outcome of the patients experienced CPR is still disappointing. Ribonuclease (RNase) has been demonstrated to have neuroprotective effects in acute stroke and postoperative cognitive impairment, possibly through acting against endogenous RNA that released from damaged tissue. However, the role of RNase in post-cardiac arrest cerebral injury is unknown. In the present study, we investigated the role of RNase in neurological outcome of mice undergoing 5 minutes of CA and followed by CPR. RNase or the same dosage of normal saline was administrated. We found that RNase administration could: 1) improve neurologic score on day 1 and day 3 after CA/CPR performance; 2) improve memory and learning ability on day 3 after training in contextual fear-conditioning test; 3) reduce extracellular RNA (exRNA) level in plasma and hippocampus tissue, and hippocampal cytokines mRNA production on day 3 after CA/CPR procedure; 4) attenuate autophagy levels in hippocampus tissue on day 3 after CA/CPR procedure. In conclusion, RNase could improve neurological function by reducing inflammation response and autophagy in mice undergoing CA/CPR.

## INTRODUCTION

Even though the cardiopulmonary resuscitation (CPR) performance is increasingly perfect, survival rate and neurological function of patients underwent cardiac arrest (CA) and CPR remain poor. Each year, around half-million people experienced sudden CA in the United States, and only 10.6% of out-of hospital CA patients survived to hospital discharge [1]. Most of the patients admitted into intensive care unit (ICU) developed severe neurological defect and other multi-organ dysfunction after return of spontaneous circulation (ROSC), which is defined as post-CA syndrome [2]. Of note, post-cardiac arrest cerebral injury is a crucial factor that results in high mortality and morbidity. Evidence has shown that cerebral injury led to a death rate of 68% among out-of-hospital CA patients, and 23% among in-hospital CA patients [3]. The underlying mechanism of post resuscitation organ dysfunction may relate to early systemic inflammatory response [4]. Previous study showed that circulating cytokines were significantly increased in non-survivors versus survivors after successful CPR [5]. Previously, there were lots of attempts to decrease whole body ischemia/reperfusion (I/R) injury after CA and CPR [6, 7]. And targeted temperature management and organ specific support are acknowledged as the effective approaches to improve neurologic outcome and survival rate of patients underwent CA and CPR [8, 9]. Abella et al. also proved that intra-arrest hypothermia could improve survival rate and neurological outcome in a mouse model of CA [10]. However, effective drug that could attenuate neurological dysfunction and improve cognition of those patients is still lacking.

Both clinical trials and basic experiments showed that systemic inflammatory response and excessive autophagy played an important role in pathophysiological processes after CA and CPR. Peberdy et al. demonstrated that early inflammatory markers, including interleukin-6 (IL-6) and interleukin-8 (IL-8) were significantly higher in out-of-hospital CA patients with poor neurologic outcome [11]. In addition, increased evidence showed that neuroinflammation played a central role in brain injury after cerebral I/R [12, 13]. Emerging evidence also showed a great importance of autophagy on determining cellular fate. Autophagy participates many physiological processes, including metabolism, aging and development [14]. However, excessive autophagy triggered by cellular stress and damage is an important pathological process leading to cell death. Previous studies have shown that excessive autophagy could result in cell death after CA and CPR [15, 16]. Preissner et al. demonstrated that extracellular RNA (exRNA), released from damaged cells, played a role in blood coagulation and vascular homeostasis [17, 18]. The authors also found that exRNA acted as a pro-inflammatory mediator and was involved in various diseases states, including inflammation and immunity process, cardiovascular diseases, neurological diseases and tumor progression [19–22]. Recent studies also found that exRNA promoted inflammatory response and apoptosis in myocardial I/R injury and lung I/R injury [23, 24]. Of note, there are scholars raised the idea that exRNA also played an important role in postoperative cognitive dysfunction (POCD) [25]. Furthermore, plasma miRNA124-3p has been reported to be associated with outcomes of patients after CA, and a reliable predictor for neurological outcome [26, 27]. Previously, ribonuclease (RNase), the counterpart of RNA, has been demonstrated to have protective effects on cardiac injury following myocardial I/R [23, 28], and pulmonary damage during lung I/R injury [24]. It is worth noting that RNase also exhibited potent neuroprotective effects on acute stroke and unilateral nephrectomy induced POCD through reducing inflammation and apoptosis [21, 29]. However, it is unknown that whether RNase treatment could attenuate neurological dysfunction and improve survival after resuscitation.

Therefore, this study was designed to investigate the effect of RNase treatment on the neurological outcome in CA/CPR mice model. We evaluated neurological condition through neurologic score and neurological behavioral test, and calculated survival rate. Furthermore, we explored the possible mechanisms of RNase treatment on neuroprotection in mice experienced CA and CPR.

## RESULTS

### Global characteristics of mice underwent CA/CPR procedure

**Figure 1:**
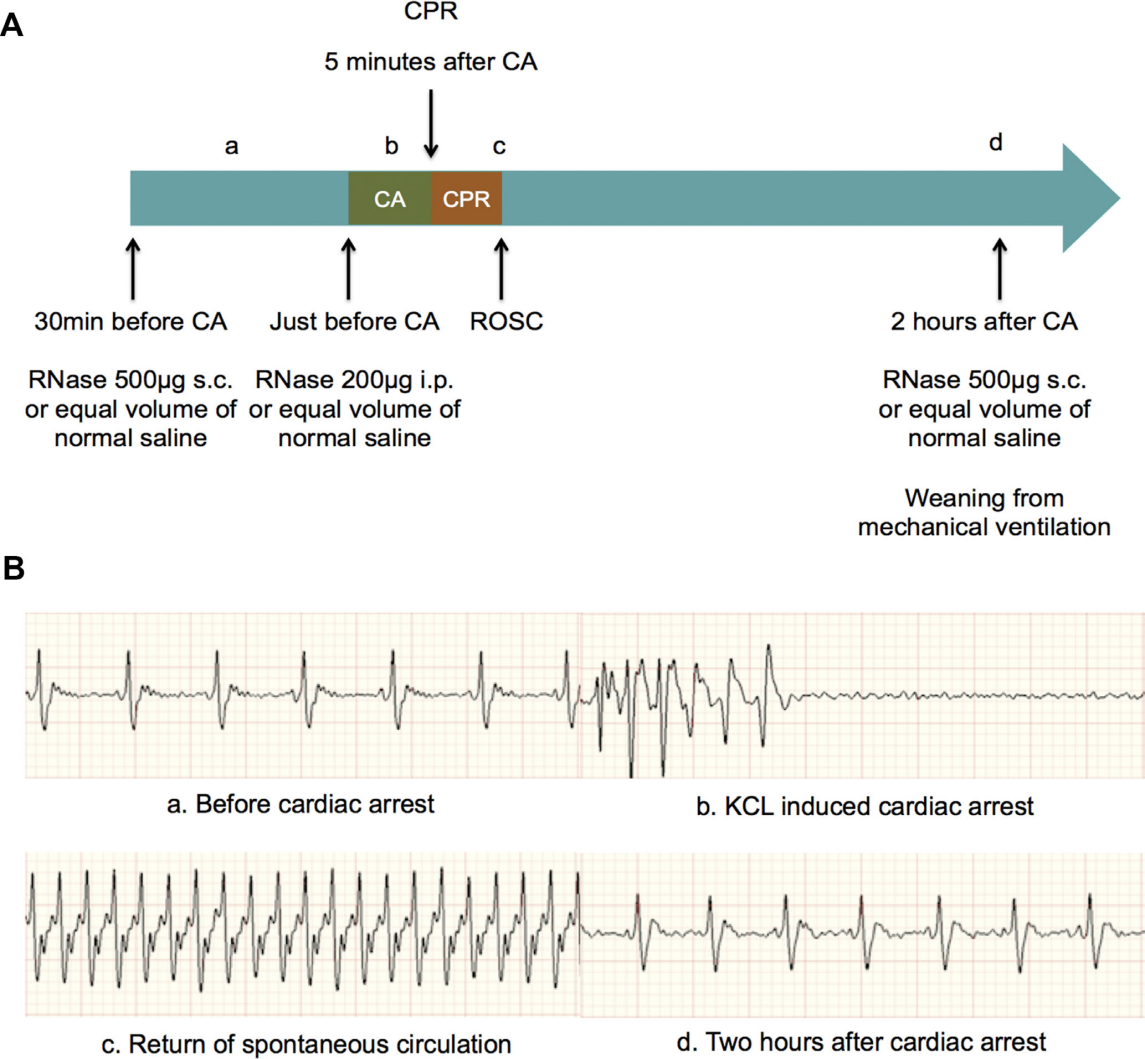
Schematic outline of cardiac arrest and cardiopulmonary resuscitation and ECGs in different stages during cardiac arrest and cardiopulmonary resuscitation procedure (**A**) Schematic outline of CA and CPR and RNase administration. (**B**) ECGs in four stages during CA and CPR: before CA (a), potassium chloride induced CA (b), ROSC (c) and two hours after CA (d). CA, cardiac arrest; CPR, cardiopulmonary resuscitation; ROSC, return of spontaneous circulation.

CA was induced by intravenous injection of potassium chloride. CPR was conducted after 5 minutes of whole body ischemia. RNase or same dosage of normal saline was given to mice. Schematic outline of CA/CPR performance and electrocardiograms (ECGs) in different stages during CA/CPR procedure were showed in Figure 1. All mice exhibited ROSC after 5 minutes of potassium chloride-induced CA followed by external chest compression, epinephrine injection and mechanical ventilation support. There were no differences for baseline characteristics between the CA/CPR plus RNase (RNCA/CPR) group and the CA/CPR plus normal saline (NSCA/CPR) group, such as body weight, body temperature and heart rate. Moreover, the time of external chest compression and dosage of epinephrine injection were similar between two groups (Table 1).

**Table 1:** Global characteristics of cardiac arrest and cardiopulmonary resuscitation procedure

	RNCA/CPR	NSCA/CPR
	*n* = 29	*n* = 29
**Baseline parameters**		
Body weight (g)	24.23 ± 0.20	24.07 ± 0.24
Body temperature (°C)	36.93 ± 0.08	36.84 ± 0.08
Heart rate before CA (bpm)	214.90 ± 13.27	226.00 ± 7.12
**Parameters of CA/CPR**		
Time to achieve ROSC (s)	89.49 ± 7.98	97.12 ± 8.08
ROSC%	100	100
Dosage of epinephrine injection (μg)	8.90 ± 0.07	8.84 ± 0.10
**Parameters after CA/CPR**		
Heart rate at 5 min after CPR (bpm)	570 ± 16	577 ± 13
Body temperature at 5 min after CPR (°C)	36.80 ± 0.06	36.92 ± 0.05
Heart rate at 1 h after CPR (bpm)	250 ± 19	267 ± 21
Body temperature at 1 h after CPR (°C)	37.01 ± 0.06	37.11 ± 0.07
Heart rate at 2 h after CPR (bpm)	412 ± 33	424 ± 27
Body temperature at 2 h after CPR (°C)	37.12 ± 0.12	37.00 ± 0.09
Ventilation time after CPR	127.30 ± 3.72	129.10 ± 2.02

Baseline parameters, parameters of CA/CPR and parameters after CA/CPR procedure were similar between RNCA/CPR group and NSCA/CPR group. CA/CPR, cardiac arrest and cardiopulmonary resuscitation; ROSC, return of spontaneous circulation; RNCA/CPR, CA/CPR plus RNase; NSCA/CPR, CA/CPR plus normal saline.

### RNase improved neurological function as well as memory and learning ability in mice underwent CA/CPR

**Figure 2:**
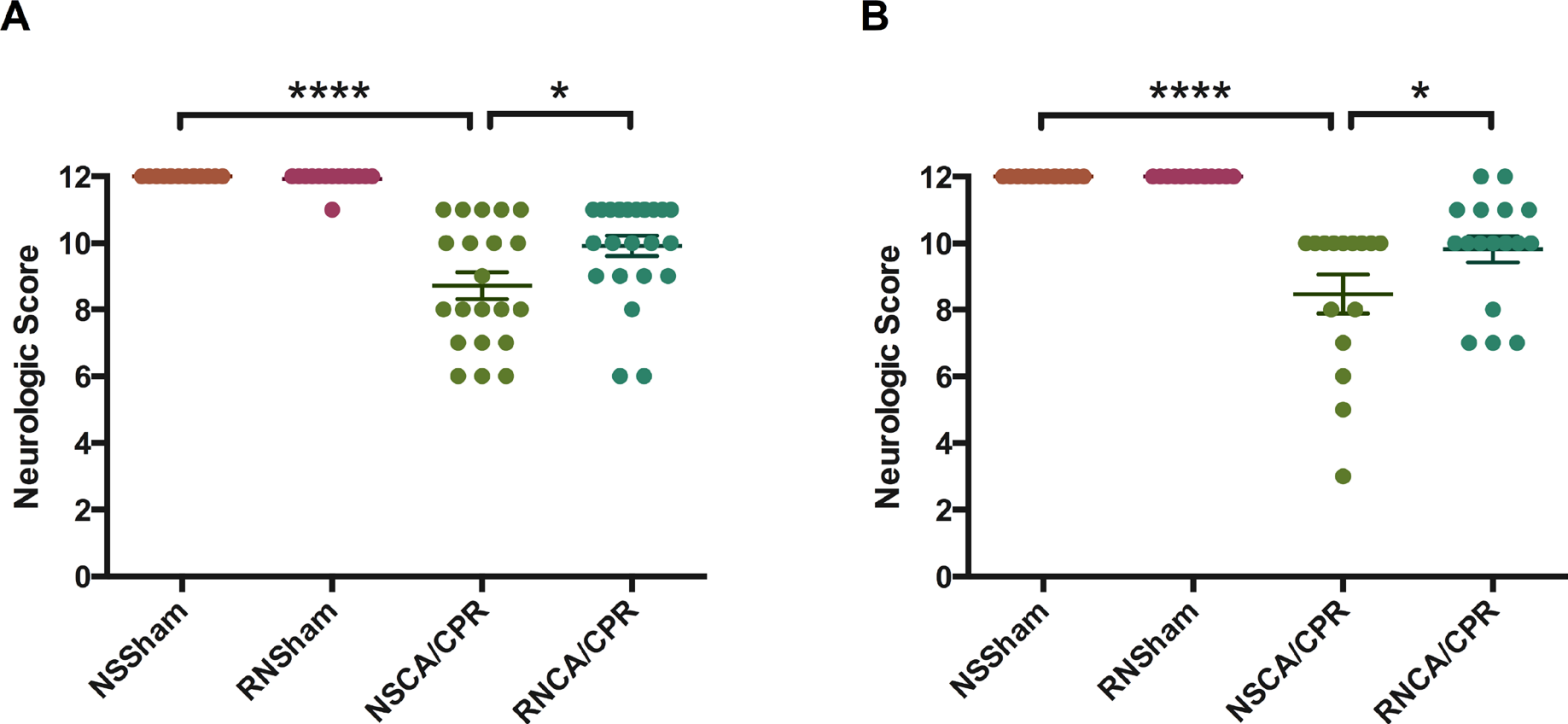
Neurologic score on day 1 (A) and day 3 (B) after successful resuscitation Neurological function was evaluated from 6 aspects: consciousness, corneal reflex, righting reflex, respiratory model, coordination and movement. Each item valued 2 points and the maximum score was 12 points. (**A**) Mean neurologic scores on day 1 after successful resuscitation in four groups. (**B**) Mean neurologic scores on day 3 after successful resuscitation in four groups. *P < 0.05, ****P < 0.0001. Data are presented as mean ± SEM (*n* = 29 per CA/CPR group, *n* = 14 per sham group). CA/CPR, cardiac arrest and cardiopulmonary resuscitation; NSSham, sham plus normal saline; RNSham, sham plus RNase; NSCA/CPR, CA/CPR plus normal saline; RNCA/CPR, CA/CPR plus RNase.

**Figure 3:**
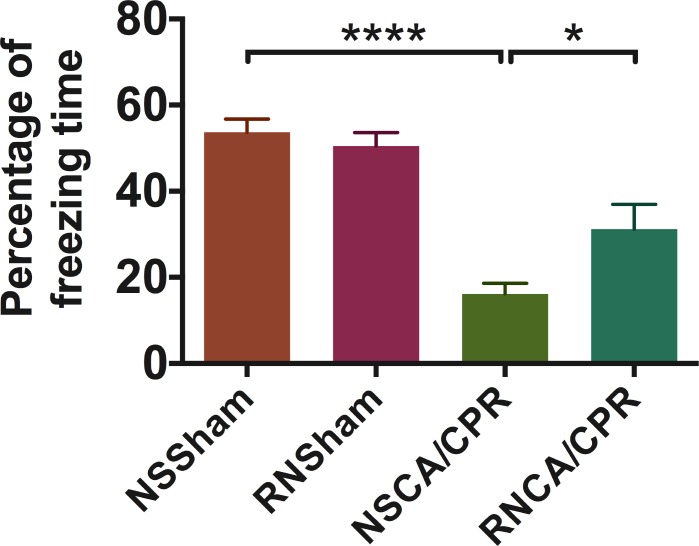
Percentage of freezing time in fear-conditioning test on day 3 after training Train conducted the day before CA/CPR performance and test conducted on day 3 after training. Total time of test process was 274 seconds. Percentage of freezing time was defined as the radio of freezing time to total time of test process. Higher value indicated better memory and learning ability. Data are presented as mean ± SEM (*n* = 8 per group). *P < 0.05, ****P < 0.0001. CA/CPR, cardiac arrest and cardiopulmonary resuscitation; NSSham, sham plus normal saline; RNSham, sham plus RNase; NSCA/CPR, CA/CPR plus normal saline; RNCA/CPR, CA/CPR plus RNase.

We evaluated neurological function of mice through 6 aspects: consciousness, corneal reflex, respiratory model, righting reflex, coordination and movement/activity. Neurologic score was significantly decreased on day 1 and day 3 after CA/CPR performance compared to sham procedure (Figure 2A, 2B, ****P < 0.0001), suggesting a significant neurological impairment due to whole-body I/R injury. RNase treatment could improve neurological outcome of mice on day 1 after CA/CPR performance (Figure 2A, 9.92 ± 0.31 vs. 8.71 ± 0.40 in the RNCA/CPR group and the NSCA/CPR group, *P < 0.05). Similarly, mice in the RNCA/CPR group had higher neurologic score compared with the NSCA/CPR group on day 3 after CA/CPR performance (Figure 2B, 9.82 ± 0.40 vs. 8.47 ± 0.58, *P < 0.05). Moreover, the neurologic score of mice in the RNCA/CPR group remained significant lower compared to that of sham surgery plus RNase (RNSham) group. In fear-conditioning test, there was a significant reduced percentage of freezing time in the CA/CPR groups compared with the sham groups. However, the RNCA/CPR group exhibited an increased freezing behavior compared with the NSCA/CPR group (Figure 3, percentage of freezing time was 32.21% ± 5.75 vs. 16.16% ± 2.51, *P < 0.05), showing a better memory and learning ability. There were no significant differences in both neurologic score and percentage of freezing time between the NSSham group and the RNSham group.

### RNase reduced cytokines expression in hippocampus tissue on day 3 after CA/CPR

**Figure 4:**
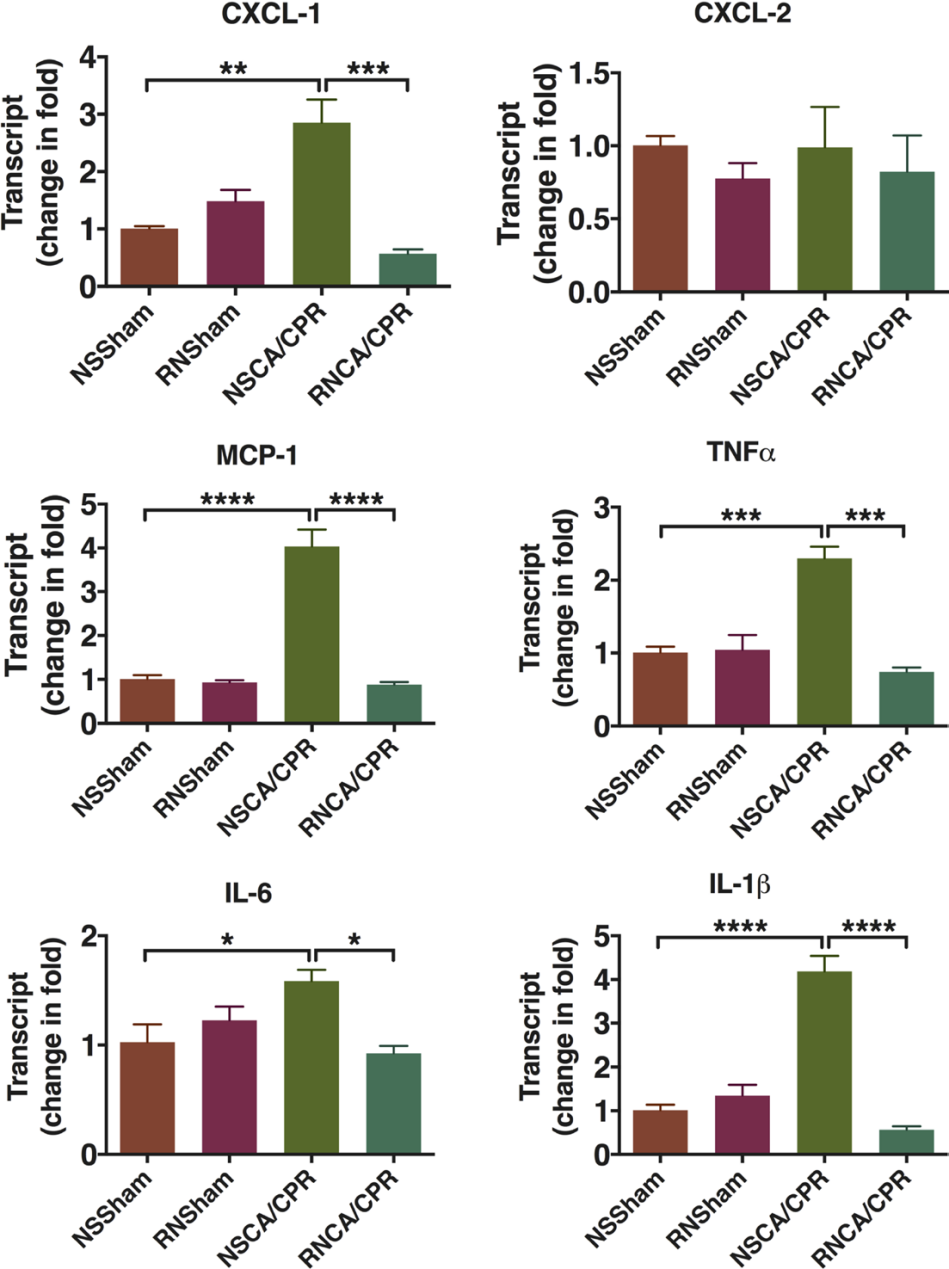
Cytokine expression in hippocampus tissue on day 3 after cardiac arrest and cardiopulmonary resuscitation Brain tissue was obtained on day 3 after modeling. Expression of cytokine mRNA in hippocampus was measured by qRT-PCR. Data are presented as mean ± SEM (*n* = 3 per group). *P < 0.05, **P < 0.01, ***P < 0.001, ****P < 0.0001. CXCL-1, chemokine (C-X-C motif) ligand 1; CXCL-2, chemokine (C-X-C motif) ligand 2; MCP-1, monocyte chemotactic protein 1; TNFα, tumor necrosis factor α; IL-6, interleukin-6; IL-1β, interleukin-1β; CA/CPR, cardiac arrest and cardiopulmonary resuscitation; NSSham, sham plus normal saline; RNSham, sham plus RNase; NSCA/CPR, CA/CPR plus normal saline; RNCA/CPR, CA/CPR plus RNase.

CA/CPR performance significantly increased the expressions of chemokine (C-X-C motif) ligand 1 (CXCL-1) and monocyte chemotactic protein 1 (MCP-1) at mRNA level in hippocampus tissue on day 3 after CPR compared to the sham groups (Figure 4, **P < 0.01, ****P < 0.0001). In addition, this increase could be significantly reduced by RNase administration (Figure 4, ***P < 0.001, ****P < 0.0001). The expressions of cytokine chemokine (C-X-C motif) ligand 2 (CXCL-2) were similar among the four groups. Inflammatory cytokines, including tumor necrosis factor α (TNFα), IL-6 and interleukin-1β (IL-1β), were also significantly increased in the NSCA/CPR group compared to that of the NSSham group. And the inflammatory cytokines were decreased significantly by RNase treatment in mice underwent CA/CPR (Figure 4, *P < 0.05, ***P < 0.001, ****P < 0.0001). There were no differences between the RNSham group and the NSSham group. This result suggested RNase administration could reduce inflammatory response on day 3 after CA/CPR.

### RNase attenuated excessive autophagy in hippocampus tissue on day 3 after CA/CPR

**Figure 5:**
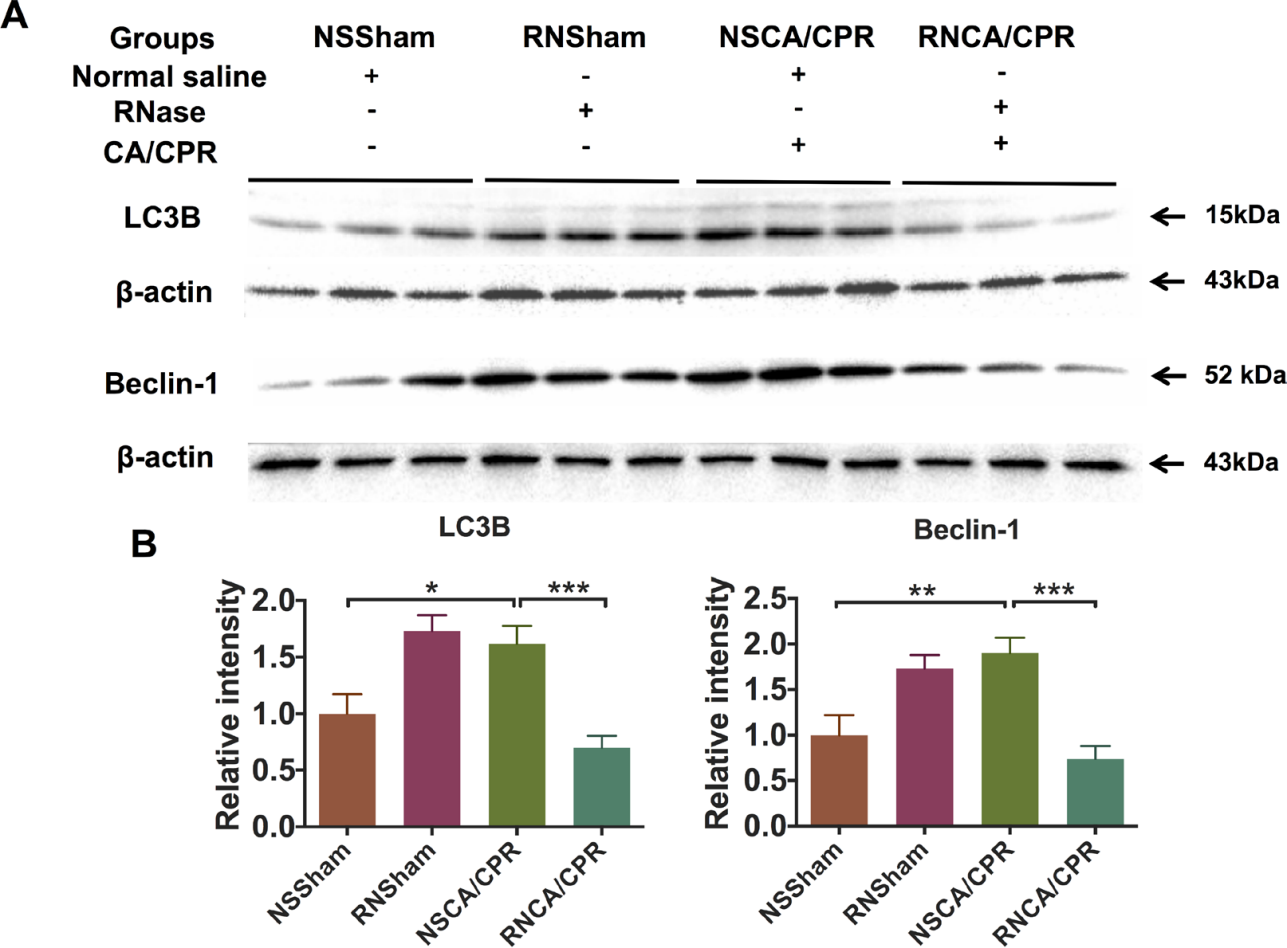
RNase treatment decreased excessive autophagy level in hippocampus tissue of mice after cardiac arrest and cardiopulmonary resuscitation (**A**) Protein levels of autophagy markers, including LC3B and Beclin-1. (**B**) Quantification of protein levels of autophagy markers. Data are presented as mean ± SEM (*n* = 6 per group). *P < 0.05, **P < 0.01, ***P < 0.001. LC3B, protein light chain-3 B; CA/CPR, cardiac arrest and cardiopulmonary resuscitation; NSSham, sham plus normal saline; RNSham, sham plus RNase; NSCA/CPR, CA/CPR plus normal saline; RNCA/CPR, CA/CPR plus RNase.

Protein level of autophagy markers, including protein light chain-3 B (LC3B) and Beclin-1, were dramatically increased on day 3 after CA/CPR in hippocampus tissue of mice compared with the NSSham group. This kind of increasing level of autophagy markers could be significantly reduced by RNase administration (Figure 5, ***P < 0.001), suggesting RNase administration could alleviate CA/CPR-induced increased level of cellular autophagy. Interestingly, autophagy markers were increased in the RNSham group compared to that of the NSSham group, indicating that RNase itself could also increase cell autophagy baseline level as an exogenous stimulation.

**Figure 6:**
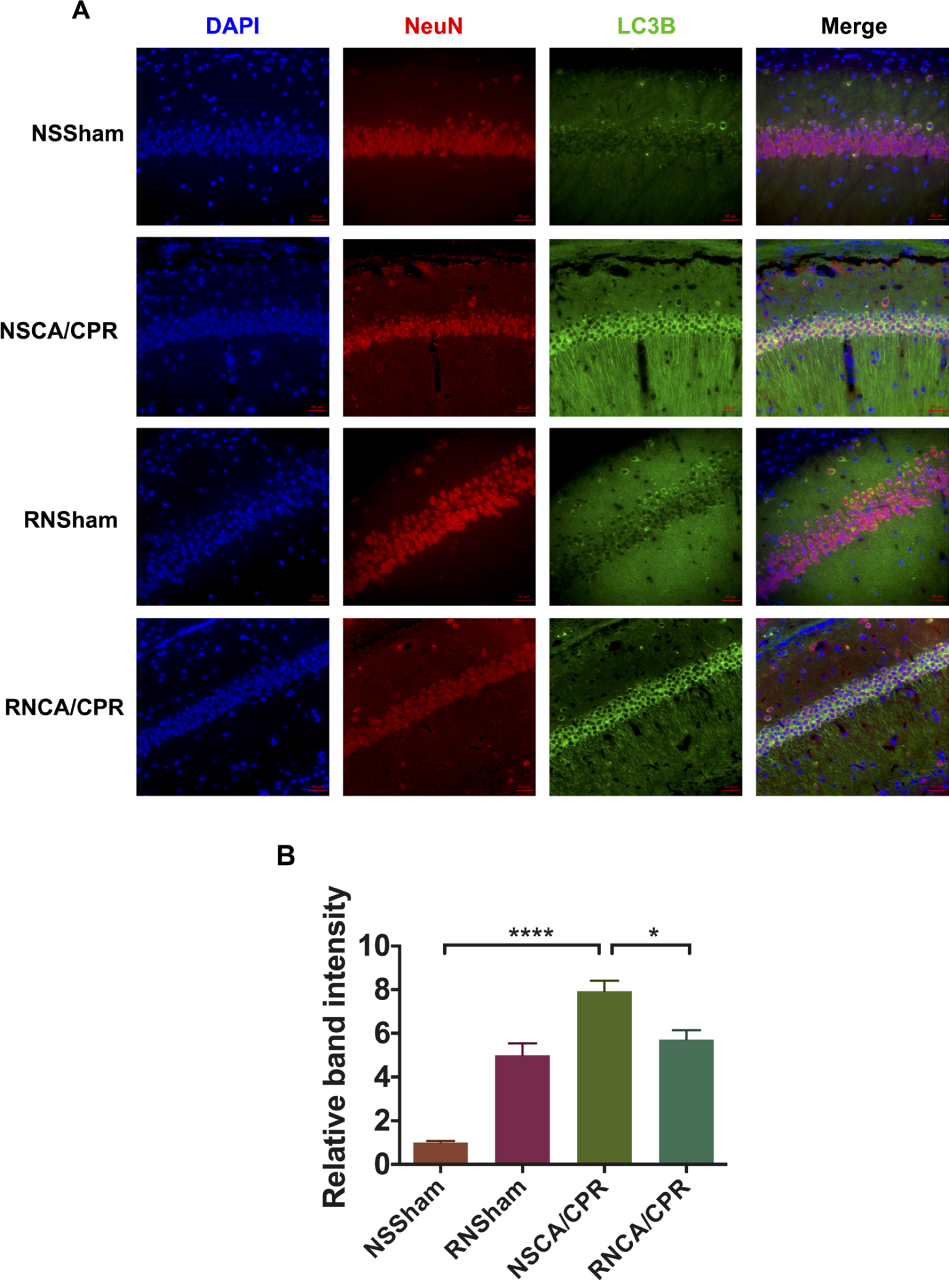
RNase treatment decreased LC3B immunofluorescent intensity in hippocampus tissue of mice underwent cardiac arrest and cardiopulmonary resuscitation (**A**) Immunofluorescent photomicrographs of hippocampus tissue in different groups. Brain was obtained on day 3 after modeling. LC3B (green) was used to indicate autophagy level in hippocampus tissue. DAPI (blue) was used to stain cellular nuclei. NeuN (red) was used to locate neurons. Pictures were obtained by fluorescence microscope. (**B**) Relative band intensity of immunofluorescence in four groups. Data are presented as mean ± SEM (*n* = 3 per group). *P < 0.05, ****P < 0.0001. LC3B, protein light chain-3 B; CA/CPR, cardiac arrest and cardiopulmonary resuscitation; NSSham, sham plus normal saline; RNSham, sham plus RNase; NSCA/CPR, CA/CPR plus normal saline; RNCA/CPR, CA/CPR plus RNase.

Immunofluorescence assays showed a higher intensity of LC3B staining in CA1 region of hippocampus tissue of mice underwent CA/CPR compared with the mice underwent sham surgery procedure, indicating excessive autophagy occurred in the NSCA/CPR group. And intensity of LC3B staining could be reduced by RNase treatment (Figure 6, *P < 0.05). Of interest, we also found that LC3B staining intensity was higher in the RNSham group compared to that of the NSSham group, which was similar to our western blot result (Figure 5).

### RNase decreased total plasma RNA concentration on day 3 after CA/CPR

**Figure 7:**
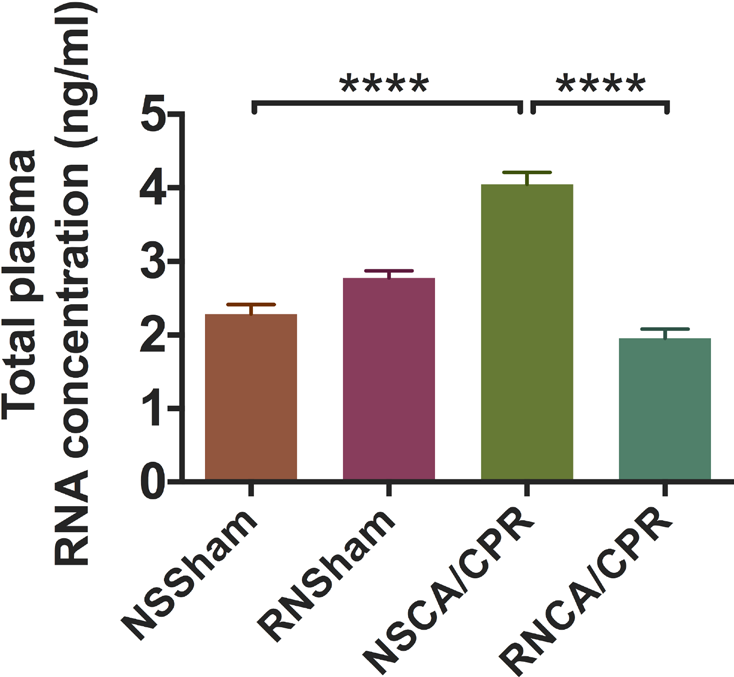
RNase treatment reduced total plasma RNA concentration on day 3 after cardiac arrest and cardiopulmonary resuscitation The concentrations of total plasma RNA were measured on day 3 after modeling in four groups. Concentrations of total plasma RNA were compared among all the four groups. Data are presented as mean ± SEM (*n* = 6 per group). ****P < 0.0001. CA/CPR, cardiac arrest and cardiopulmonary resuscitation; NSSham, sham plus normal saline; RNSham, sham plus RNase; NSCA/CPR, CA/CPR plus normal saline; RNCA/CPR, CA/CPR plus RNase.

Mice whole blood were collected on day 3 after modeling. Plasma total RNA was extracted and quantified under a stringent RNase-free condition. The total plasma RNA concentration was significantly increased in the NSCA/CPR group compared with the NSSham group (Figure 7, ****P < 0.0001). RNase treatment significantly decreased total plasma RNA concentration in the RNCA/CPR group compared with the NSCA/CPR group (Figure 7, ****P < 0.0001). Total plasma RNA concentration was similar between the RNCA/CPR group and the NSSham group. These results suggested RNase administration might play a protective role in neurological function of mice undergoing CA/CPR through reduction of total plasma RNA level.

### RNase reduced exRNA level in hippocampus tissue on day 3 after CA/CPR

**Figure 8:**
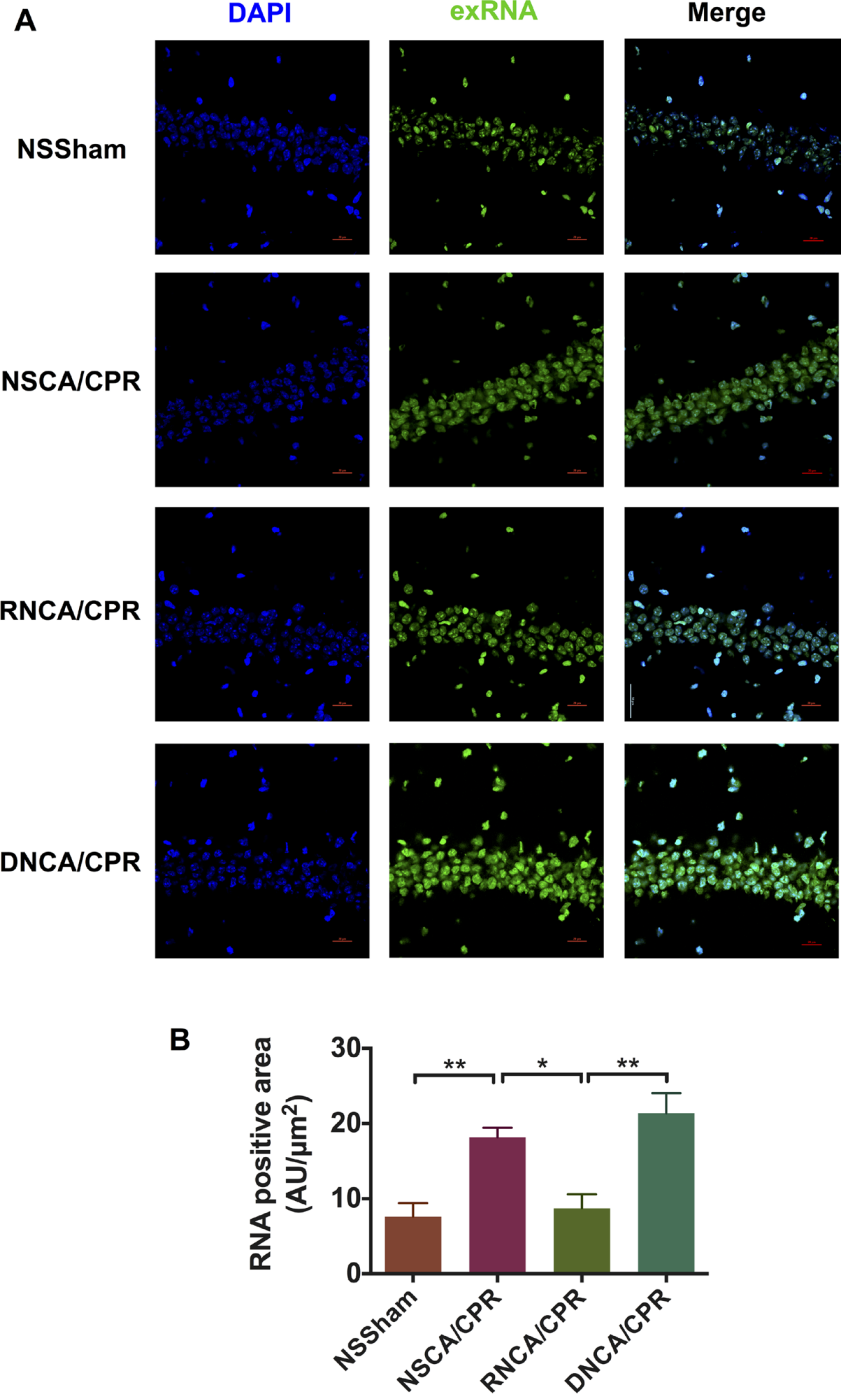
Staining and quantitative analysis of exRNA-associated fluorescence in mouse hippocampus CA1 area on day 3 after cardiac arrest and cardiopulmonary resuscitation (**A**) Distribution of exRNA in hippocampus CA1 area of mice in different groups. Brain tissue was obtained on day 3 after CA/CPR. The exRNA was stained by SYTO^®^ RNASelect^™^ Green Flourescent Cell Stain (green), and cell nuclei were stained by DAPI (blue). Images were taken by confocal microscope. (**B**) Quantitative analysis of fluorescence intensity of exRNA in hippocampus CA1 area. Data are presented as mean ± SEM (*n* = 3 per group). *P < 0.05, **P < 0.01. CA/CPR, cardiac arrest and cardiopulmonary resuscitation; NSSham, sham plus normal saline; RNSham, sham plus RNase; NSCA/CPR, CA/CPR plus normal saline; RNCA/CPR, CA/CPR plus RNase; DNCA/CPR, CA/CPR plus DNase.

To clearly demonstrate whether the beneficial effect of RNase is due to its extracellular enzymatic function to degrade extracellular RNA, we stained RNA by using RNASelect^™^ Stain and showed the RNA distribution in the hippocampus CA1 area of mice in four groups (Figure 8A). We calculated the RNA-positive area and found that mice in the NSSham group showed low exRNA level in hippocampus tissue. The exRNA levels were similar among sham groups, including the NSSham group, the RNSham group and sham surgery plus DNase (DNSham) group (data not show). Of note, we found that exRNA level was significantly increased after CA/CPR (with normal saline treatment) compared with the NSSham group (Figure 8B, **P < 0.01). In addition, RNase had significantly reduced the exRNA level in hippocampus tissue of mice on day 3 after CA/CPR procedure (Figure 8B, *P < 0.05). The results showed that RNase functioned as a cerebral protector after CA/CPR by degrading exRNA in hippocampus tissue. Considering that the total plasma RNA level also decreased significantly in mice underwent CA/CPR with RNase treatment, RNase in our protocol indeed reduced exRNA in both peripheral circulation and central nervous system.

### Effects of RNase administration on survival rate after CA/CPR

**Figure 9:**
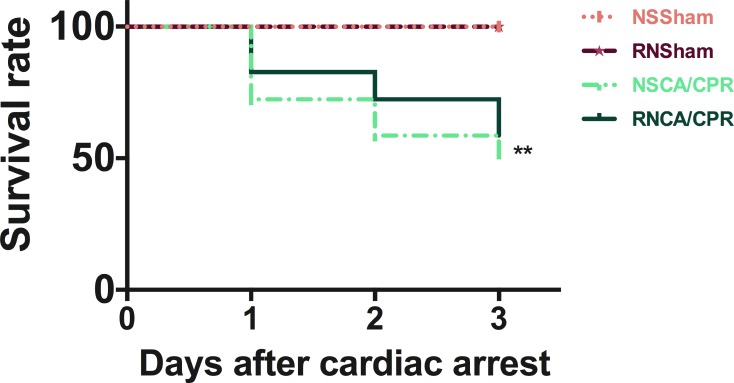
Survival rates within 3 days in different groups Observation period was 3 days after CA/CPR procedure (*n* = 29 per CA/CPR group, *n* = 14 per sham group). **P < 0.01, NSCA/CPR group vs. NSSham group. CA/CPR, cardiac arrest and cardiopulmonary resuscitation; NSSham, sham plus normal saline; RNSham, sham plus RNase; NSCA/CPR, CA/CPR plus normal saline; RNCA/CPR, CA/CPR plus RNase.

The total numbers of mice in the NSSham group and the RNSham group were both 14. All of them were survived till day 3 after sham surgery. The total numbers of mice in the NSCA/CPR group and the RNCA/CPR group were both 29. In the RNCA/CPR group, The numbers of mice survived from CA/CPR procedure on day 1, day 2 and day 3 were 24, 21 and 17, respectively. In NSCA/CPR group, there were 21 mice survived on day 1 after CA/CPR procedure, 17 mice on day 2 after CA/CPR and 15 left on day 3 after CA/CPR. Survival rates on day 1 after CA/CPR were 82% and 74% in the RNCA/CPR group and the NACA/CPR group respectively. On day 2 after CA/CPR, survival rates went down to 72% and 58% in these two groups respectively. And on day 3 after CA/CPR, respective survival rates decreased to 58% and 51% in the RNCA/CPR group and the NSCA/CPR group (Figure 9). Unexpectedly, we only found a higher survival trend in the RNCA/CPR group, but failed to reach statistical significance compared with the NSCA/CPR group.

## DISCUSSION

In the present study, we investigated the role of RNase in neurological function after 5 minutes of CA and followed by CPR performance in a mice model. We found that CA and CPR performance could result in severe neurological impairment, which could be significantly reduced by RNase treatment. Moreover, RNase administration could decrease the level of plasma RNA concentration, exRNA level and inflammatory cytokine expression in hippocampus tissue, and inhibit excessive autophagy in hippocampus tissue induced by CA/CPR. Also, our study revealed an increasing trend of survival rate in the RNCA/CPR group compared with the NSCA/CPR group, however, not statistically different. The 3-day survival rate was 51% in the NSCA/CPR group and 58% in the RNCA/CPR group.

There are some methodological considerations of our experiment. First, in our settings, 5 minutes of CA exhibited a marked abnormality both in neurologic score and in contextual fear-conditioning test, and increased inflammatory response as well as excessive autophagy level in the hippocampal tissue, although this CA time period seemed short. We demonstrated that the survival rate in the NSCA/CPR group was 51%, which was quantitatively similar to that of the previous studies [30, 31]. In our pilot study, we used potassium chloride-induced 5 and 8 minutes CA model. We found that mice underwent 5 minutes of CA has a moderate mortality with about 50% mice survived on day 3 after CA/CPR. To investigate long-term neurological outcome of mice survived from CA/CPR performance, we chose 5 minutes of CA period that could provide a potential window of opportunity. Second, considering clinical settings that hypothermic treatment was not included in out-of hospital CPR procedure, we did not cool down the mice during CA and CPR period. This may lead to a higher mortality compared to other studies.

After successfully achieving ROSC, cerebral and myocardial dysfunction, systemic I/R injury and persist pathology lead to high mortality and morbidity induced by whole-body I/R injury, which is called post-cardiac arrest syndrome [2, 4]. Post-cardiac arrest syndrome is a kind of sepsis-like syndrome through activation of inflammatory response after whole-body I/R. Bro-Jeppesen et al. revealed that IL-6 was independently associated with increased mortality of patient underwent out-of hospital CA [32]. Moreover, in a CA/CPR swine model, increased pro-inflammatory cytokines expression, such as IL-1β and TNFα, were shown after resuscitation and played an important role in post-cardiac arrest syndrome [33]. Importantly, hydrogen-rich saline treatment could reduce the production of inflammatory cytokines, such as TNFα and IL-1β, after CA/CPR procedure so that it could improve survival condition and neurological outcome of rats [34]. Therefore, these studies indicated that reducing inflammation level might be a potential therapy to improve clinical outcome.

Endogenous damage-associated molecular pattern molecules (DAMPs), released from stressed, necrotic and damaged cells, could initiate innate immune reaction and promote inflammatory response. DAMPs, such as heat-shock proteins (HSPs), uric acid, high-mobility group box protein 1 (HMGB1), S100 proteins, DNA, RNA and ATP [35], has been revealed involving lots of pathological process, like I/R injury [36], atherosclerotic plaque formation [20], persistent pain [37] and tumor growth [38]. Of note, exRNA, one of the DAMPs, has been demonstrated to play a detrimental role in myocardial and lung I/R injury through mediation of inflammation and apoptosis [23, 24]. Also, previous study has shown that circulating miRNA, miRNA-122 and miRNA-21, were overexpressed in CA/CPR patients with poor neurological outcome [39]. In the present study, we have found that RNase administration could also improve outcome after CA and CPR through reduction of the exRNA level in both plasma and hippocampus tissue. The underlying mechanism may also be related to the inhibition of pro-inflammatory cytokines production, as cytokines, such as CXCL-1, MCP-1, IL-1β, IL-6, and TNFα were significantly reduced after RNase treatment on day 3 after CA and CPR.

Autophagy is an important intracellular physiological process of degrading mis-folded proteins and damaged organelles to maintain cellular homeostasis and survival [40]. It is a kind of cytoprotection against stress and damage. A research proved that autophagy in cerebral I/R process played a neuronal protective role by mitophagy-related mitochondria clearance [41]. In addition, dysfunction of autophagy is related to many neurodegenerative diseases [42]. On the other hand, emerging evidence has shown that excessive autophagy played a crucial role in cell death during cerebral ischemia [43–45], as well as CA and CPR process [15, 16, 46]. Thus, autophagy is a double-edged sword and has been regarded as a main modifier of neuronal survival or death [47]. In our present study, we found that excessive autophagy level was significantly inhibited by RNase administration, as demonstrated by decreased expression of protein levels of LC3B and Beclin-1 in western blot analysis, as well as LC3B staining intensity in immunofluorescence analysis in the RNCA/CPR group compared to that of the NSCA/CPR group. To our surprise, we also discovered that these autophagy markers were dramatically increased in the RNSham group, compared with the NSSham group. This could be explained based on the following study. Microinjection of RNase A into human fibroblasts could increase rate of uptake of protein in lysosomal pathway [48]. The study indicated that the administration of RNase A could also increase baseline level of autophagy in cells. Thus, we infer that RNase A administration could induce autophagy process in hippocampal tissue of mice undergoing sham procedure in a physiological way. Furthermore, we didn't find RNase treatment had any impact on the neurologic score and neurological behavioral test in the sham group. Therefore, RNase may exhibit neuroprotective role through inhibition of the deleterious autophagy process activated in brain after CA/CPR.

Admittedly, our study also has some limitations. First, RNase A has a low molecular weight and a short half-life time in plasma, so that RNase could be cleared via kidney rapidly [49]. Continuously administer RNase could be a more effective way to digest exRNA. This may be the reason why the administration protocol of RNase in our study failed to increase survival rate but just improve neurological function after CA/CPR. In addition, some kinds of exRNA could be protected from digestion by microvesicles or RNA-binding proteins. In our present study, what kind of degree had exRNA been digested and what specific kind of exRNA played the most important role in CA/CPR process are still unclear. Second, the way of RNase administration in this study was based on previously published studies. However, as the unpredictable onset of sudden CA, the rescue window is relatively narrow. Post-conditioning is much more valuable under this circumstance. Further study is required to investigate the most suitable dosage and timing of RNase administration. Third, extended period of observation time is needed in future study to figure out the long-term prognosis of mice. Expectedly, survival rate may reach statistical difference in the RNCA/CPR group compared to the NSCA/CPR group as time goes on. Fourth, we collected blood and brain tissue only on day 3 after modeling. Analysis of total plasma RNA and exRNA in hippocampus tissue, inflammatory response and autophagy level at more time points may help to understand the detailed pathological progress better. In conclusion, RNase plays an important role in attenuating neurological dysfunction after CA/CPR. RNase could reduce exRNA level and systemic inflammation response, as well as excessive autophagy in mice model of CA/CPR. RNase may be a candidate agent against post-CA cerebral injury. However, the detailed signaling mechanisms should be explored in further studies.

## MATERIALS AND METHODS

### Experimental animals and grouping

Male C57BL/6J mice with body weight of 23–25 g and age of 8–12 w were purchased from Dashuo experimental animal company (Chengdu, China). Animals were raised in 12 hours’ light/dark cycle condition and free to laboratory water and food. Our protocol has been approved by Animal Care and Use Committee of Sichuan University (No.2015025A). This study was conducted with supervision of this committee and all efforts were made to minimize suffering of mice. Mice were randomized into four groups: NSSham, RNSham, NSCA/CPR group and RNCA/CPR group. We also involved three mice in the DNSham group and three mice in the DNCA/CPR group for exRNA expression analysis.

### Cardiac arrest and cardiopulmonary resuscitation procedures

Protocol of CA/CPR procedure was based on previous studies [30, 50, 51] and modified according to the result of our pilot study. Mice were anesthetized by intraperitoneal (i.p.) injection of 100 μg/g ketamine and 10 μg/g xylazine and then placed on a heating plate. Intubation with a 22-gauge intubation cannula and mechanical ventilation (Harvard Bioscience, USA) with respiratory frequency of 130 beat per minute (bpm), tidal volume of 10 μl/g and fraction of inspired oxygen (FiO_2_) of 0.4, as previous published protocol. Body temperature was monitored with a rectal temperature probe (Indus instruments, USA) during the whole procedure and maintained the body temperature at 37 ± 0.5°C. Needle probe ECG (Indus instruments, USA) was monitored throughout the whole procedure. A heparinized micro-PE catheter (PE10) was inserted into right external jugular vein under microscope for drug administration.

CA was induced by bolus of 80 μg/g potassium chloride through micro-PE catheter. In the meantime, mechanical ventilation was interrupted. CA was confirmed through non-electrical activity appearance in ECG. Resuscitation was initiated after 5 minutes of CA at a frequency of 350–400 bpm using index finger. At the same time, ventilation was resumed with respiratory frequency of 150 bpm, FiO_2_ of 1.0 and 0.4 μg/g epinephrine was given through micro-PE catheter. ROSC was confirmed through ECG with regular spontaneous electrical activity and visual heart beat at chest. Respiratory frequency was reduced to 130 bpm and FiO_2_ was reduced to 0.4 at 20 minutes after initiation of CPR. Micro-PE catheter was removed at 1 hour after ROSC and incision was sutured. Mice weaned from mechanical ventilation at 2 hours after ROSC (Figure 1). Needle probe ECG and rectal temperature probe were removed just before extubation. Ropivacaine (0.2%, 50 μl) was given to prevent post-operative pain.

RNCA/CPR group received three doses of filtrated RNase A (ThermoFisher, USA): 500 μg/100 μl, subcutaneous (s.c.), 30 minutes before CA; 200 μg/200 μl, i.p., right before CA; 500 μg/100 μl, s.c., 2 hours after CA as previously described [23, 24, 29]. RNSham group received RNase A in the same way. NSCA/CPR and NSSham group received equal volume of normal saline at the same time points. Drug was injected in a blinded fashion. Mice in RNSham and NSSham group only undergoing anesthesia, intubation, mechanical ventilation, skin incision, insertion of micro-PE catheter and post-operative analgesia.

### Neurological function test

#### Neurologic score

Neurological function was evaluated from the following 6 aspects as previously published method [10, 52]: consciousness (response to tail pinch), corneal reflex, and respiration model, righting reflex, coordination and movement/ activity. Within each item, mice could get 0, 1 or 2 according to different conditions and total score was 12 maximally. Neurologic score was assessed by an observer blinded to the treatment.

### Fear-conditioning test

Fear-conditioning test was used to evaluate memory and learning ability in rodents. Mice were trained the day before CA/CPR procedure as previous described protocol [53]. Mice were transported into behavioral test room and left undisturbed for 20 minutes. Each mouse was put into a conditioning chamber (Ugo Basile, Varese, Italy) exploring in context for 100s followed by conditional stimulus (20s-cue tone, 75dB, 5 kHz) and unconditional stimulus (2s-foot shock, 0.75mA). This procedure was repeated once time and mouse was removed 30 s later. Total training time was 274 s. Fear-conditioning test was conducted on day 3 after training as previous published protocol [53]. Mice were transported into training room and left undisturbed for 20 minutes. Mouse was placed into the same conditioning chamber for 274 s with context exploration and without cue tone and foot shock. Freezing behavior showed a better memory ability of aversive memory of foot shock. We calculated percentage of freeing time to evaluate the memory and learning ability of mice in the four groups.

### Quantification of total plasma RNA concentration

Mouse whole blood was collected on day 3 after CA/CPR procedure in RNase-free Eppendorf tubes. Whole blood was centrifuged in 4°C Cryogenic centrifuge at 4000 rpm for 10 minutes. Supernatant plasma was collected in RNase-free Eppendorf tubes. We used 200 μl plasma to extract (Eastep Universal RNA Extraction Kit) and quantify (Thermo Scientific NanoDrop 2000 spectrophotometer) total plasma RNA. All the procedures were performed under a stringent RNase-free environment.

### Quantification of exRNA level in hippocampus tissue

On day 3 after CA/CPR or sham surgery procedure, mouse brain tissue was collected after anesthesia, put into liquid nitrogen as soon as possible, and kept at −80°C. Frozen brain tissue was cut into 30 μm-thick frozen sections. Frozen sections were fixed with methanol for 1 hour at 4°C and then washed by PBS. Brain frozen sections were stained by SYTO^®^ RNASelect^™^ Green Flourescent Cell Stain (S32703, Molecular Probes, USA) for 20 minutes at 37°C and washed by PBS. DAPI was used to stain cellular nuclei. Confocal microscope (A1RMP+, Nikon, Japan) was used to detect fluorescent intensity of exRNA in hippocampus tissue with three filters under high magnification (600×). Quantification the exRNA staining was performed in three microscopic fields (600×) each section in a blinded way.

### Quantification of cytokines mRNA

Mouse hippocampus tissue was collected on day 3 after CA/CPR procedure. qRT-PCR was performed to quantification of cytokines mRNA expression in hippocampus as previously described [29]. RNA from hippocampus tissue was extracted with TRIzol reagent. Primer sequence used for qRT-PCR as previously described [23, 29]. qRT-PCR was performed on Eppendorf PCR system. 18s RNA level was used to normalize data in every sample.

### Western blot analysis

Protein was extracted as previously described [54]. Equal volume (100 μg) of protein was resolved on a 15% SDS-PAGE and transferred to polyvinylidene difluoride membrane. The membranes were intubated overnight at 4°C with primary antibodies, including anti-LC3B (192890, Abcam, England), anti-Beclin-1 (207612, Abcam, England) and anti-β-actin (TA-09, ZSGB, China) antibody. Membranes were intubated with second antibodies, including goat anti-rabbit IgG antibody (2B-2301, ZSGB, China) and goat anti-mice IgG antibody (2B-2305, ZSGB, China). Immunoreactivity was detected using ECL fluorescent detection reagent. Density of immunoreactive bands was analyzed by NIH Image J software. β-actin acted as a loading control.

### Immunofluorescent analysis

For detection of autophagy in hippocampal tissue, immunofluorescent analysis was conducted. Under deep anesthesia, mice were perfused with cold PBS, followed with 2% paraformaldehyde and 2.5% glutaraldehyde. Paraffin-embedded brain tissue was cut into 10-μm-thick coronal brain sections. Brain sections were treated with primary antibodies against LC3B (ab192890, Abcam, England) and NeuN (MAB377, Millipore, USA) overnight. Primary antibodies were cleared with PBS, and brain sections were intubated with secondary antibodies: Alexa Fluor^®^ 488 donkey anti-mouse IgG (H+L) (A21202, ThermoFisher, USA) and Alexa Fluor^®^ 647 donkey anti-rabbit IgG (H+L) (A31573, ThermoFisher, USA). DAPI was used to stain cellular nuclei. The immunoreactivity was detected with a fluorescence microscope (Axio imager Z2, Carl Zeiss, Germany) with three filters under high magnification (400×). Quantification the immunoreactivity of LC3B staining was performed in three microscopic fields (400×) each section in a blinded way.

### Statistical analysis

Statistical analysis was conducted on GraphPad Prism 7 software in a blinded fashion. All data were expressed as means ± SEM. Log-rank test was used to test data for Kaplan-Meier Survival Analysis. Student t test was used to analyze mean differences between two groups. One-way analysis of variance (ANOVA) was used to analyze differences among four groups. *P* < 0.05 was considered to be statistically different.

## Abbreviations

ANOVA: one-way analysis of variance
bpm: beat per minute
CA: cardiac arrest
CXCL-1: cytokine chemokine (C-X-C motif) ligand 1
CXCL-2: cytokine chemokine (C-X-C motif) ligand 2
CPR: cardiopulmonary resuscitation
DAMPs: damage-associated molecular pattern molecules
DNCA/CPR: CA/CPR plus DNase
DNSham: sham surgery plus DNase
ECG: electrocardiogram
exRNA: extracellular RNA
FiO_2_: fraction of inspired oxygen
HMGB1: high-mobility group box protein 1
HSPs: heat-shock proteins
ICU: intensive care unit
IL-1β: interleukin-1β
IL-6: interleukin-6
IL-8: interleukin-8
i.p.: intraperitoneal
I/R: ischemia/reperfusion
LC3B: protein light chain-3 B
MCP-1: monocyte chemotactic protein 1
MI: myocardial infarct
miRNA: microRNA
NSCA/CPR: CA/CPR plus normal saline
NSSham: sham surgery plus normal saline
PVDF: polyvinylidene difluoride
RNase A: ribonuclease A
RNCA/CPR: CA/CPR plus RNase
RNSham: sham surgery plus RNase
ROSC: return of spontaneous circulation
s.c.: subcutaneous
TNFα: tumor necrosis factor α
